# Proteomic analysis of murine testes lipid droplets

**DOI:** 10.1038/srep12070

**Published:** 2015-07-10

**Authors:** Weiyi Wang, Suning Wei, Linghai Li, Xueying Su, Congkuo Du, Fengjuan Li, Bin Geng, Pingsheng Liu, Guoheng Xu

**Affiliations:** 1 Department of Physiology and Pathophysiology, School of Basic Medical Sciences, Peking University, Beijing 100191, China; 2 National Laboratory of Biomacromolecules, Institute of Biophysics, Chinese Academy of Sciences, Beijing, 100101, China

## Abstract

Testicular Leydig cells contain abundant cytoplasmic lipid droplets (LDs) as a cholesteryl-ester store for releasing cholesterols as the precursor substrate for testosterone biosynthesis. Here, we identified the protein composition of testicular LDs purified from adult mice by using mass spectrometry and immunodetection. Among 337 proteins identified, 144 were previously detected in LD proteomes; 44 were confirmed by microscopy. Testicular LDs contained multiple Rab GTPases, chaperones, and proteins involved in glucuronidation, ubiquination and transport, many known to modulate LD formation and LD-related cellular functions. In particular, testicular LDs contained many members of both the perilipin family and classical lipase/esterase superfamily assembled predominately in adipocyte LDs. Thus, testicular LDs might be regulated similar to adipocyte LDs. Remarkably, testicular LDs contained a large number of classical enzymes for biosynthesis and metabolism of cholesterol and hormonal steroids, so steroidogenic reactions might occur on testicular LDs or the steroidogenic enzymes and products could be transferred through testicular LDs. These characteristics differ from the LDs in most other types of cells, so testicular LDs could be an active organelle functionally involved in steroidogenesis.

The testis consists of three major cell types: germ cells, Sertoli supporting cells within seminiferous tubules, and Leydig cells in the interstitium between the tubules. Leydig cells are particularly enriched with endoplasmic reticulum (ER), mitochondria, and cytoplasmic lipid droplets (LDs)^1^,^2^. This structure is associated with the androgen production function of Leydig cells.

Testosterone biosynthetic enzymes are generally located in the ER and mitochondrial membranes and the adjacent cytoplasm. The precursor substrate for steroidogenesis is cholesterol. An individual Leydig cell could secrete 20 ng of testosterone daily in humans^3^ and 0.5 ng in adult rodents^2^. To ensure such a high rate of steroidogenesis, the testis utilizes endogenous cholesterols *de novo* synthesized *in situ* rather than transported from the plasma^4^,^5^. The intracellular LDs of Leydig cells contain a large pool of cholesteryl ester that can be broken down into free cholesterol on demand for steroidogenesis^5^. In response to the varied androgen production during pubertal growth^6^ and breeding^1^, the size and number of LDs in Leydig cells may vary greatly, which reflects an altered demand for stored cholesterol-cholesteryl ester for testosterone biosynthesis^1^,^6^. Also, Sertoli cells contain a fair amount of small LDs that show cyclic variations throughout the spermatogenic cycle in rat^7^ and human^8^ and can transfer from Sertoli cells to spermatocytes^8^. Therefore, testicular LDs play functional roles in testes.

The LDs in all eukaryotes contain a core of neutral lipids, a monolayer surface of phospholipids, and a number of proteins that are embedded in the surface^9^. In contrast to biochemically inert neutral lipids, the protein components on the LD surface are biologically active and control LD storage and hydrolysis and LD-related cellular functions. A considerable number of LD proteins have been identified in many types of cells by immunodetection or proteomic approaches. The investigation of these LD proteins has greatly extended our understanding of the properties and functions of LDs in given cells.

The LDs in testicular cells are particularly small, with mean diameter 1 μm2, and thus are not easily detected by common immunodetection approaches. Only a few LD-associated proteins have been identified in testicular cells. This insufficient information has long restricted the investigation of functional roles of testicular LDs.

This proteomic study aimed to identify protein components of testicular LDs of adult mice. We detected 337 proteins from testicular LD preparations; 144 were prevously detected in LD proteomes and 44 were previously verified in LDs by microscopy. Testicular LDs contained almost complete sets of LD-related protein members of both the perilipin (Plin) family and lipase/esterase superfamily that assemble predominantly in adipocyte LDs and contain many enzymes that govern biosynthesis of sterols and hormonal steroids. These distinct characteristics are different from the LDs in most other cells. Testicular LDs are a unique, biologically active cellular organelle that might be regulated like adipocyte LDs and play important roles in the biosynthesis and metabolism of hormonal steroids.

## Methods and Materials

### Animals and antibodies

Polyclonal antibodies against Plin1~4 and hormone-sensitive lipase (HSL) were from C. Londos (US National Institutes of Health). Other antibodies were from Abcam, Cell Signaling, or Santa Cruz Biotechnology. The animal study was performed in accordance with the NIH guidelines for the care and use of laboratory animals and was approved by the animal care and utilization committee of Peking University Health Science Center.

### Purification of the LDs from mice testis

For each individual preparation, 20 testes obtained from 10-week-old C57BL/6 mice were used. LDs were purified by the protocol we developed recently^10^. Manipulations were performed at 4 °C or on ice, if required. After removal of blood vessels and connective tissues, 20 testes were grouped and homogenized by use of a Dounce glass homogenizer containing 10 ml buffer A (250 mM sucrose, 0.2 mM phenylmethylsulfonyl fluoride, 25 mM tricine, pH 7.6) by 20 strokes with a loose-fitting pestle and 40 strokes with a tight-fitting pestle. The homogenate was disrupted for 15 min at 750 psi in a nitrogen bomb chamber and cleaned by centrifugation at 3000 × g. The post-nuclear supernatant was transferred to a SW40 tube, then buffer B (20 mM HEPES, pH 7.4, 100 mM KCl and 2 mM MgCl_2_) was loaded on top of the supernatant. After centrifugation at 38,000 × g for 1 h, a white LD layer appeared on the top of the tube. The membrane was pelleted at the bottom, and the infranatant was the cytosolic fraction. All 3 fractions were collected. The LD fraction was transferred to a new tube and centrifuged for 4 min at 14,000 × g. After removal of the underlying liquid, LDs were washed 3 times, each with 200 μl buffer B and centrifuged at 14,000 × g for 4 min. The LD fraction on the top was collected.

### Protein in-gel digestion and mass spectrometry analysis

Manipulations were performed as we reported recently^11^. Protein components in the LD preparation were precipitated with 100% acetone. Proteins were separated by 10% SDS-PAGE followed by Coomassie Blue or silver staining. For the total proteome, a full lane of Coomassie Blue-stained gel was cut into 23 slices from high to low molecular weight. Each slice was further cut into smaller pieces, destained, washed, dehydrated and vacuum-dried. Proteins in slices were reduced with 10 mM dithiothreitol for 1 h at 56 °C and alkylated with 55 mM iodoacetamide for 45 min. Gel slices were washed with 25 mM ammonium bicarbonate, acetonitrile and vacuum-dried. For in-gel digestion, slices were incubated with 10 ng/μl trypsin in 25 mM ammonium bicarbonate solution. The digestion reaction proceeded at 37 °C overnight and was stopped by adding 5% formic acid to adjust pH to <4.0. After two extractions with 60% acetonitrile, the tryptic peptide mixture was vacuum-dried and dissolved in 0.1% formic acid. Peptide extracts were purified on a C18 trap column and analyzed by use of a 2D-HPLC system coupled to a linear ion-trap mass spectrometer (Thermo Fisher Scientific, MA).

### Immunoblotting

Proteins from the LD preparation were extracted with acetone, separated by 10% SDS-PAGE, and underwent immunoblotting analysis with primary antibodies, then horseradish peroxidase-conjugated lgG. The blots were developed with enhanced chemiluminescence detection reagents (Applygen Technologies, Beijing).

### Histology and immunofluorescence

Mice testes were fixed with 4% paraformaldehyde and embedded in paraffin and cut. For routine histology, sections were stained with hematoxylin-eosin. For immunofluorescence staining, sections were incubated for 10 min with 3% H_2_O_2_ to eliminate endogenous peroxidase activity and underwent antigen retrieval with 0.3% sodium citrate and phosphate buffered saline, pH 7.4, for 15 min at 72 °C. Sections were blocked with 1% defatted albumin and immunostained with primary antibody, then FITC-labeled lgG. Signals were observed under a Nikon Eclipse 50i fluorescence microscope.

### LD staining

LDs in frozen testicular sections were stained with Nile Red. Nuclei were stained with Hoechst 33258. For *in vitro* staining, LDs purified from testicular tissue were spread on glass slides, dried, and stained with Lipid-TOX Deep Red. Fluorescent signals were viewed under an Olympus FV1000 confocal microscope.

### Thin-layer chromatography

LDs were purified from brown adipose tissue and testes of mice and from cultured Chinese hamster ovary (CHO) cells. Total lipids in different LD preparations were extracted in chloroform and acetone (1:2, v/v) and centrifuged at 14,000 × g for 10 min. The organic phase was collected and dried under nitrogen gas. Lipid extracts were dissolved in chloroform and loaded on silica gel plates for analysis. Neutral lipids were separated on plates in a hexane:diethyl ether:acetic acid (80:20:1, v/v/v) solvent system and visualized by the iodine vapor method.

### Data mining and bioinformatics

To obtain reliable results, we performed at least two biological replicates of proteomic analysis and results were combined for further analysis. The online database used to sort the proteomic table was http://genome.ucsc.edu/cgi-bin/hgNear. Protein associations were revealed by the Website program String (http://string-db.org/).

## Results

### Testicular LD staining

**Figure 1:**
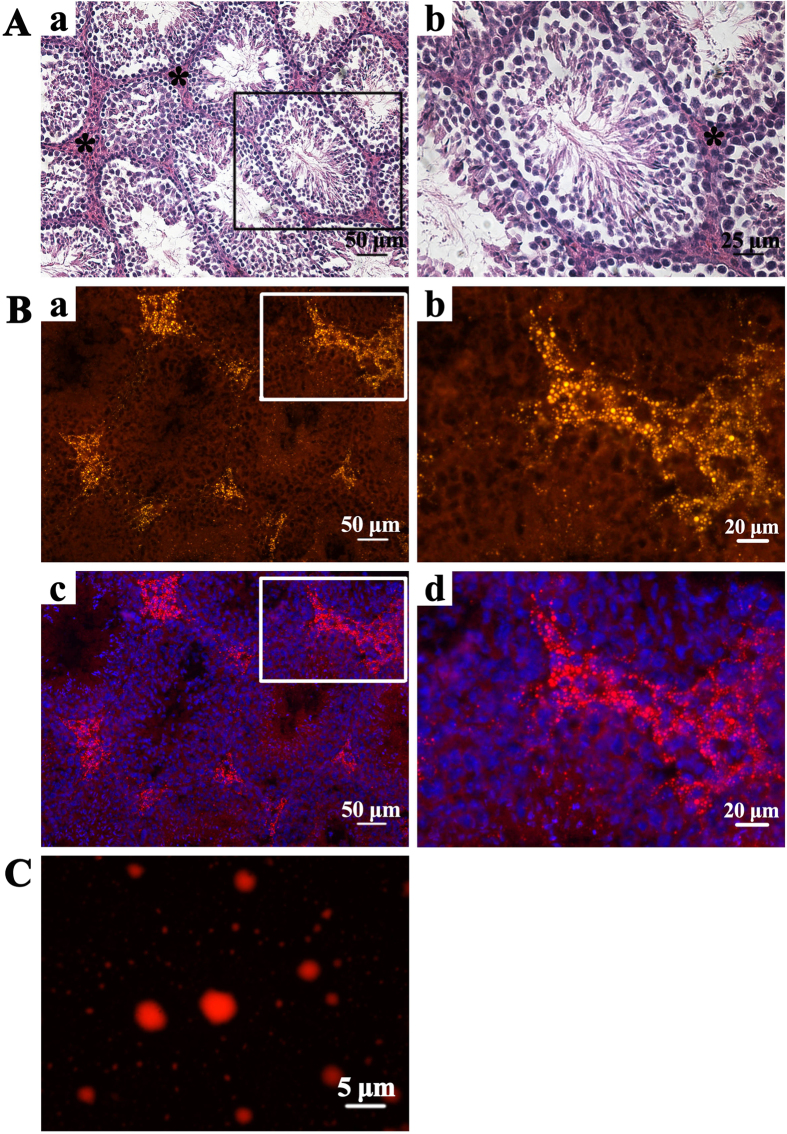
Testicular lipid droplets (LDs) staining **A**. Hematoxylin-eosin staining of mouse testicular tissue. The asterisk marks the interstitium between the seminiferous tubules in panel a. The amplified images of boxed area are in panel b. **B**. LDs stained with Nile Red in frozen testis sections. Nuclei were stained with Hoechst 33258. Panel a and b, Nild Red stained LDs. Panel c and d, the merged images. **C**. LDs purified from mice testes were spread on slides and stained with Lipid-TOX Deep Red.

Interstitial cells were located in the interstitium between the seminiferous tubules of mouse testicular tissue (Fig. 1A, panel a and b). Numerous small, concrete LDs stained with Nile Red were observed in interstitial Leydig cells rather than in the cells located within the seminiferous tubules (Fig. 1B). Lipid-TOX staining showed that the LDs prepared for proteomic analysis were morphologically intact, with a diameter of about 1 μm, despite the presence of a few large droplets (Fig. 1C).

### Lipid and protein patterns of testicular LDs

**Figure 2:**
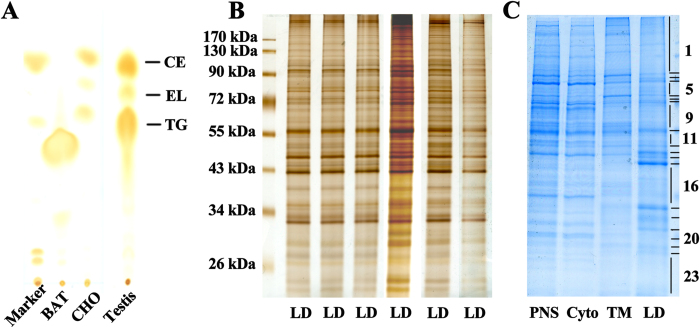
Lipid and protein patterns in testicular LDs **A**. Thin-layer chromatography analysis of total lipids extracted from LD preparations of mice testis, brown adipose tissue (BAT), and Chinese hamster ovary (CHO) cells. TG, triacylglycerols; CE, cholesteryl esters; EL, ether lipid. **B**. Silver-stained SDS-PAGE gels of protein extracts of different testicular lipid droplet preparations. **C**. Coomassie Blue-stained SDS-PAGE gels of proteins extracted from fractions of testicular LD, total membrane (TM), cytosol (Cyto), and post-nuclear supernatant (PNS). For the whole proteome, the lane running the testicular LD proteins was excised into 23 gel slices and underwent mass spectrometry.

Thin-layer chromatography revealed that mice testicular LDs consisted of a fairly equivalent amount of cholesteryl esters and triacylglycerols and a small amount of ether lipid, similar to steroidogenic CHO cells; by contrast, adipose LDs contained a large amount of triacylglycerols but few cholesteryl esters and ether lipid (Fig. 2A). Equal amounts of protein extracted from different compartments were separated by SDS-PAGE. Silver staining revealed that the proteins in different LD preparations showed a highly consistent band pattern in gels (Fig. 2B), which indicated the reliability of the LD purification. In contrast, the protein band pattern of LD fractions differed from that of total membrane, cytosol, and post-nuclear supernatant fractions (Fig. 2C).

### Proteomic analysis of testicular LD proteins

**Figure 3:**
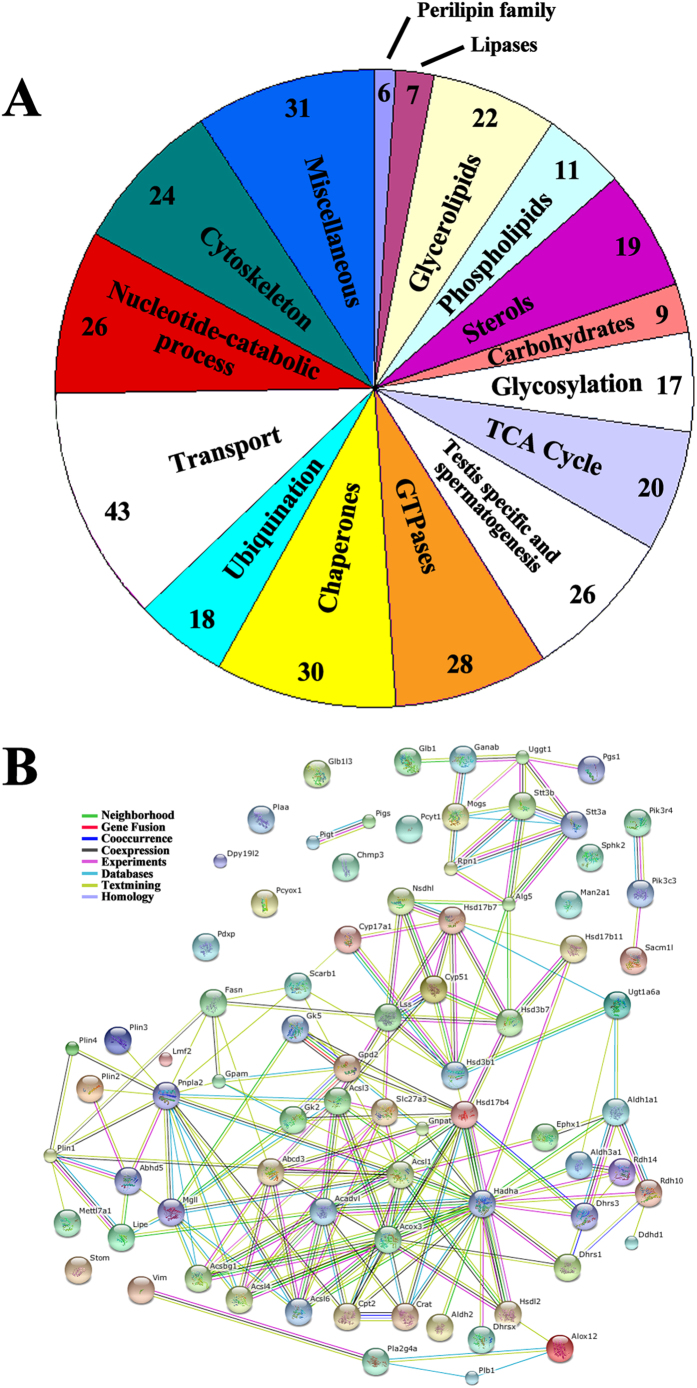
Properties of murine testicular LD proteins **A**. Protein categories of mouse testis LDs. All proteins identified by 2D-LC MS/MS were sorted by subcellular distributions and known functions based on literature or NCBI online sources. **B**. Network of function-related LD proteins. Lines in different colors represent functional association in various types of evidence. Red, fusion evidence; green, neighborhood evidence; blue, co-occurrence evidence; purple, experiment evidence; yellow, text-mining evidence; black, co-expression evidence.

For the whole proteome of testicular LDs, the lane running testicular LD protein was excised into 23 gel slices (Fig. 2C). After in-gel digestion, tryptic peptides underwent mass spectrometry analysis. Only proteins with at least two unique peptides were accepted for identification. A total of 337 proteins were identified; at least 144 (42.7% of total) were previously reported in LD proteomes of other mammalian cells or tissues and 44 were previously confirmed in LDs by microscopy. Each identified protein and its encoding gene were searched in the UniProt and NCBI databases and PubMed. The 337 proteins were classified into 16 groups by known or putative functional annotation for identified proteins (Fig. 3 and Table 1).

Group 1 proteins represented vimentin^12^ and stomatin^13^ and particularly Plin1, Plin2/ADRP, Plin3/Tip47, and Plin4/S3-12, 4 classical LD proteins belonging to the perilipin family of 5 LD proteins (Plin1~5) conserved in their first ~100 amino-terminal residues^9^. Plin1 binds and links vimentin to LDs, then vimentin filaments wrap the LDs tightly in a cage-like spherical structure surrounded by multiple ER tubules, thus facilitating LD formation^12^. Plin2~4 widely express and localize at LDs and non-LD compartments, but Plin1 expresses exclusively in adipose and steroidogenic cells and localizes only at the LD surface^9^. Plin1~4 provide a barrier and protect LDs against access by HSL and adipose triglyceride lipase (ATGL), but native Plin1 is more protective than Plin2~4^14^,^15^. Interestingly, testicular LDs contained 4 variants of Plin1, termed Plin1a, 1b, 1c, and 1d, which share conserved N-terminal 198 residues and 11-mer regions. This was the first identification of Plin1d protein in the tissue (Table 1).

Group 2 included 7 lipases/esterases/thioesterases, which cover almost all currently known cellular lipases/esterases. HSL^14^, ATGL and its co-lipase CGI-58 represent more than 95% of the lipolytic activity in adipocytes^16^, with the remaining hydrolase activity contributed by triacylglycerol hydrolase/carboxylesterase 3^17^,^18^ and monoglyceride lipase^19^,^20^. LD-associated hydrolase (C2orf43 protein) is a cholesteryl ester hydrolase that normally localizes to the ER but is translocated to LDs on lipid loading^21^,^22^. ATGL expresses specifically in adipose tissue^23^, but HSL expresses primarily in both adipose and steroidogenic tissues.

Group 3 proteins represented 22 enzymes involved in the metabolism of fatty acid and glycerolipids and as well as phospholipids and sterols. Five were previously observed in LDs by microscopy. Fatty acid transport protein 1 binds diacylglycerol acyltransferase 2 and colocalizes to the ER-LD interface to facilitate glycerolipid biosynthesis and LD expansion^24^. Long-chain acyl-CoA synthetase Acsl1^25^ and Acsl3^25^,^26^, along with glycerol-3-phosphate O-acyltransferase (Gpat4), are normally localized in the ER microdomain but effectively translocated to nascent LDs to facilitate LD biosynthesis on lipid loading^25^,^26^. Acsl4 and fatty aldehyde dehydrogenase were morphologically localized in yeast LDs^27^ and proteomically detected in LDs of CHO cells^28^, adipocytes^29^ and mouse muscle^30^. Carnitine O-palmitoyltransferase 2, very-long-chain acyl-CoA dehydrogenase, and mitochondrial trifunctional enzyme subunit α were detected from mouse muscle LDs^30^. Fatty acid synthase was detected from LDs of granulosa steroidogenic cells from rat ovary^20^. Many proteins in this group are known to specifically or highly express in testes (Table 1).

Group 4 proteins represented 11 phospholipid metabolic enzymes; 3 were previously physiologically confirmed in LDs. Phospholipase B is highly expressed in testis and activated by sterol removal in murine sperm membrane, which localizes at the LD surface and hydrolyzes glycerophospholipids to facilitate the LD structure^31^. Cytosolic phospholipase A2 (cPLA2) is activated by extracellular stimuli-hydrolyzed arachidonic acids from the sn-2 position of glycerophospholipids; in turn, released arachidonic acids induce the translocation of cPLA2 to the ER and LD interface to regulate lipid synthesis and nascent LD formation^32^,^33^. Phosphocholine cytidylyltransferase binds to growing LDs and then catalyzes phospholipid synthesis and promotes LD expansion^34^,^35^. Phosphatidylglycerophosphate synthase 1 and phosphoinositide lipid phosphatase are highly expressed in testes, and phospholipase DDHD1 is required for spermatogenesis. The proteins in this group also participate in glycerolipid and sterol metabolism.

Group 5 contained 19 proteins that participate in biosynthesis and metabolism of cholesterol, retinol, and hormonal steroids; 6 were previously observed in LDs by microscopy and another 7 were previously detected in LD proteomes. Short-chain dehydrogenase/reductase 3 and retinol dehydrogenase 10 are reciprocally activated and on acyl ester biosynthesis, are translocated from the ER to LDs^36^,^37^,^38^. The key steroidogenic enzymes lanosterol synthase^27^, 3β-hydroxysteroid dehydrogenase (HSD) 1 and 7^30^,^39^,^40^, 17β-HSD-4, −7, −11 and −17^11^,^30^,^40^,^41^,^42^, and NAD(P)H steroid dehydrogenase-like^43^,^44^ were previously microscopically or proteomically detected in intracellular LDs. Many of these enzymes, such as 17-α-hydroxyprogesterone aldolase and scavenger receptor class B-I^20^, are highly expressed in testes and regulate cholesterol homeostasis.

Group 6 proteins represented 17 enzymes involving in glucuronidation and glycosylation. UDP-glucuronosyltransferase 1–6^40^, DolP-glucosyltransferase^11^,^28^, α-glucosidase^20^, and methyltransferase-like protein 7A^40^ were previously found in LD proteomes, and methyltransferase-like protein 7B was observed in LDs by microscopy^19^,^45^,^46^. CGI-49 proteins are frequently found in LD proteomes^11^,^29^,^30^,^41^. Large oligosaccharyltransferase complexes contain ribophorin I, Stt3a, Stt3b, p97/Vcp, Sel1l, and Ubxd8^47^ and may also interact with ancient ubiquitous protein 1 (Aup1), Acsl3 and stomatin^48^. Ubxd8^49^,^50^, p97/Vcp^49^,^50^, Aup1^48^, Acsl3^25^,^26^ and stomatin^13^ have been verified in LDs by microscopy, which suggests that the present identification is reliable. Several enzymes in this group catalyze glucuronidation reactions of estrogens, testosterones, retinoic acids, and various metabolites of xenobiotics and endobiotics^47^.

Group 7 and 8 proteins included 29 enzymes involved in the metabolism of carbohydrate and tricarboxylic acid cycle. NADH-cytochrome b5 reductase was verified in LDs by microscopy^45^. Glutamate dehydrogenase, malate dehydrogenase, succinate dehydrogenase, lactate dehydrogenase, pyruvate kinase 2/3, and citrate synthase were previously reported in LD proteomes^11^,^20^,^51^. The identification of 17 other metabolic enzymes in testicular LDs is novel, which might reflect the close relationship between LDs and mitochondria in testicular cells^52^.

Group 9 proteins represented 28 small GTPases; 27 were previously reported in LD proteomes. In cells loaded with fatty acids, Rab5a^53^, Rab11a^53^, Arl2 GTPase Elmod2^54^, and Rab18^53^,^55^ can localize to both the ER and LDs, where Rab18 recruits unknown effectors and microtubules to facilitate membrane trafficking and lipid exchange^53^,^55^. Testicular LDs might serve as a dock for various small GTPases for mediating Rab signaling.

Group 10 listed 30 protein chaperones; 18 were previously reported in LD proteomes. We previously showed that heat shock protein 70 (Hsp70) can translocate to adipocyte LDs on heat stimulation^56^. Spermatid-specific Hsp70, Hsp70.2 (Hspa2), T-complex protein 11, and protein disulfide-isomerase A3 (PDI3a) are testis-specific and play roles in spermatogenesis. PDI is a component of microsomal triacylglycerol transfer protein complex. T-complex protein 1 contains 8 distinct subunits to form a unique chaperone for escorting actin, tubulin, and numerous other proteins. In Leydig cells, the intermediate filaments of the cytoskeletons may bind to LDs^52^.

Group 11 listed 18 proteins involved in proteasome and membrane trafficking. Among them, p97, Atad3a and Afg3l2 are AAA ATPase family proteins that regulate ubiquination, membrane trafficking, and organelle biogenesis. p97, Ubxd2 and Ubxd8/Faf2) bind with each other and colocalize to LDs^46^,^49^,^50^. Aup1 localizes to the ER and LDs^48^,^57^. Aup1 may exist in several subcomplexes and associate with numerous other proteins^48^ such as Ubxd8, Ubxd2, Atad3a, RuvB-like 1, stomatin, ribophorin I and II, T-complex proteins, epoxide hydrolase 1, atlastin-3, Acsl3, pyruvate kinase 2/3, PDI, and ATP synthase^48^. Dozens of Aup1-associated proteins were also identified in testicular LDs, which might reflect the close association of these protein complexes with cellular LDs.

Group 12 contained 43 transport proteins; 16 were proteomically reported^11^,^30^,^40^,^58^ and 5 were microscopically confirmed in LDs^59^,^60^. Coatomer protein complex I (COPI) and clathrin adaptor complex mediate intra-Golgi transport and retrograde transport from the Golgi to ER. Arf1/COPI complexes localize between the ER and LDs for targeting the triacylglycerol synthesis enzyme Gpat4 to the LD surface and bud 60-nm nanodroplets from the LDs. In cells loaded with fatty acids, both COPI and COPII (Sec23) structures tend to localize to discrete foci surrounding LDs to create a membrane bridge for transporting ATGL and Plin2 to nascent LDs^60^.

Group 13 contained 26 proteins involved in nucleotide-catabolic processes, such as ion transport, transcription, translation, and cell signaling. Nine proteins were detected by previous LD proteomes. Some proteins might not easily fit into this single category because of the divergence of protein functions. MAPK/ERK kinase 2 is colocalized with cPLA2 in LDs, then rapidly activates cPLA2 for releasing arachidonate from LDs^33^; it is required for testosterone synthesis in Leydig cells. ATP synthase subunit α and sodium pump subunit α1 and α4 are expressed abundantly in testis and regulate spermatogenesis.

Group 14~16 included cytoskeletal proteins, testis-specific and miscellaneous proteins. Only 11 of these 81 proteins were previously reported in LD proteomes. The identification of albumin in the present and previous LD proteomes should represent a contamination because of its abundance in serum. The identification of testis-specific proteins could be due to the contamination or the difficult separation of these protein components from testicular LDs. For example, GAPDH2 and A-kinase anchor protein 3 and 4 participate in spermatogenesis, which can bind the cytoskeletal fibrous sheath and thus might be co-purified with LD-associated cytoskeletons. Also, these testis-specific or spermatogenesis-related proteins might exist in cellular subcomplex structures that associate with testicular LDs^52^.

### Confirmation of testicular LD protein identification by immunoblotting and immunofluorescence

**Figure 4:**
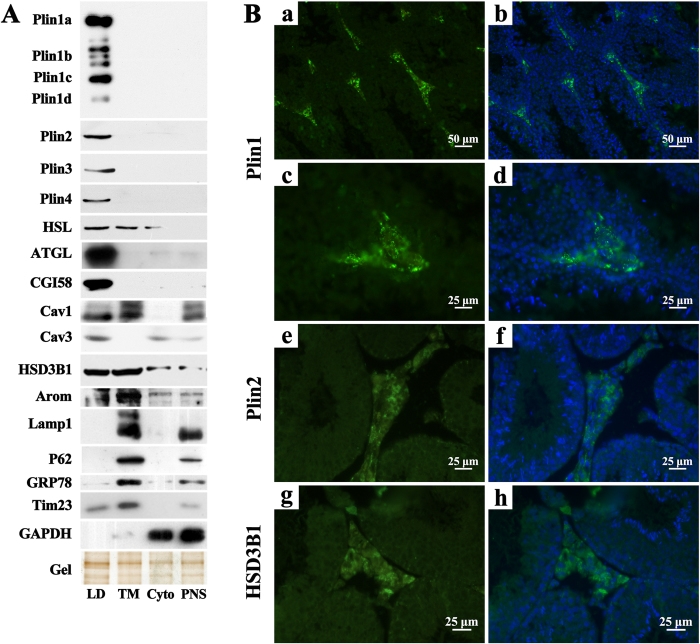
Confirmation of LD proteins by immunodetection **A**. The fractions of LD, total membrane (TM), cytosol (Cyto), and post-nuclear supernatant (PNS) were prepared from mice testes. An equal amount of proteins extracted from different fractions was separated by SDS-PAGE and underwent immunoblotting with the primary antibodies indicated. A representative silver-stained gel showed equivalent protein loading. Plin variants Plin1a~1d were detected on a full-length blot. The blots of proteins were derived from the sample or different samples that were processed in parallel, and the corresponding full-length blots are shown in Supplementary Figure S1. Arom, aromatase; Cav, caveolin; Plin, perilipin; HSL, hormone-sensitive lipase; ATGL, adipose triglyceride lipase. **B**. Immunofluorescent staining of Plin1 (**a,c**) Plin2 (**e**), and 3β-hydroxysteroid dehydrogenase (HSD3B1) (**g**) in sections of mouse testis. The merged images are shown in panel b,d,f and h.

Some of the identified testicular LD proteins were confirmed by immunoblotting by using marker proteins corresponding to different cellular compartments (Fig. 4A). Four members of the perilipin family, Plin1~4, including the 4 variants of Plin1, Plin1a, 1b, 1c and 1d, were detected only in the LD fraction. This was the first immunodetection of Plin1d in tissue (Fig. 4A). Plin5 signal was not detectable in testicular LD extracts (data not shown), which is consistent with its low level of expression in non-oxidative tissues. ATGL and CGI-58 appeared only in the LD fraction; HSL and 3β-HSD1 were highly enriched in the LD fraction but also detectable in the membrane and cytosol compartments (Fig. 4A). Caveolin-1 and -3, caveolae marker proteins, were not identified in the testicular LD proteome (Table 1) but were immunodetected in the LD fraction or other cellular compartments (Fig. 4A). Aromatase, a cyp19 enzyme that converts androgen to estrogen in seminiferous epithelium, was marginally detected in the testicular LD fraction but appeared mainly in the membrane fraction (Fig. 4A). Lysosome protein Lamp-1, ER protein p62, and cytoplasmic enzyme GAPDH were not detected in the LD fraction. The ER chaperone GRP78 and mitochondrial protein Tim 23 were detected predominately in the membrane and post-nuclear supernatant fractions, but a small amount appeared in the LD fraction (Fig. 4A). Clearly, the isolated LD fraction of mice testes was largely free of other organelle contamination, although a small amount of the ER and mitochondria components might be introduced, likely because of their abundance or general interactions with LDs^61^. Furthermore, immunofluorescent signals of Plin1 appeared strongly in the interstitium of mice testis (Fig. 4B, panel a and c), and the fluorescent signal pattern was consistent with that of interstitial LDs stained with Nile Red (Fig. 1B, panel a and b). Immunofluorescent signals were weaker for Plin2 and 3β-HSD1 than Plin1 but still detectable in interstitial locations (Fig. 4B, panel e and g). The immunofluorescent signal for 17β-HSD11 was not detected (data not shown).

## Discussion

We report the first proteomic analysis of LDs purified from adult mice testes. Testicular LDs contained 337 proteins; 144 were previously detected in LD proteomes and 44 were verified by microscopy. From the functions of identified proteins, testicular LDs showed several characteristics different from the LDs of most other cell types. Testicular LDs may be unique, biologically active cellular organelles that might have functional roles in the biosynthesis of hormonal steroids.

First, testicular LDs featured most Plin family and lipase/esterase superfamily proteins and various enzymes for biosynthesis and metabolism of glycerolipids and phospholipids. The classical LD proteins, Plin1~4 and 4 variants of Plin1, are crucial for regulating LD formation^9^. During LD expansion in differentiating adipocytes, nascent small LDs are coated with Plin3 and Plin4, medial-size LDs require both Plin2 and Plin1, and finally, Plin1 replaces Plin2 as a major coat of large LDs in mature adipocytes^62^. We previously revealed that Plin2 is degraded by the proteasome with the induction of Plin1^63^ and if Plin1 is null for replacing Plin2, LD growth and adipocyte differentiation are retarded^64^. Different Plins target different types of LDs and have unique functions to govern triacylglyceride–cholesterol ester balance^15^. Plin1a and Plin1b favor triacylglyceride-rich LDs^15^, Plin1c and Plin4 prefer cholesteryl ester-rich LDs, but Plin2 and Plin3 show less specific localization to LDs^15^. Plin1 expresses exclusively in adipose and steroidogenic cells^9^. Thin-layer chromatography revealed that the LD of adipocytes was triacylglyceride-rich, so it associates mainly with Plin1a and Plin1b. In contrast, the testicular LD had a relatively equivalent proportion of triacylglycerides and cholesteryl esters. The accumulation of triacylglycerides promotes and stabilizes storage of cholesteryl esters within Leydig cells^5^. Likely, the coats of Plin1~4, including Plin1a~1d, could cooperatively manipulate the appropriate balance of cholesteryl ester-triacylglycerides in steroidogenic cells of testes.

Also, testicular LDs contained most of the known lipases/esterases/phospholipases and enzymes of glycerolipid and phospholipid metabolism. HSL and ATGL represent ~95% of the lipolytic activity in adipocytes^16^ and the remaining activity is contributed by triacylglycerol hydrolase^17^,^18^ and monoglyceride lipase^19^. We and others previously revealed that Plin1 phosphorylation induces the translocation of HSL from the cytosol to LDs^14^,^65^ and also indirectly activates ATGL by unsequestering the ATGL coactivator CGI-58, hence conferring a full lipolytic reaction in adipocytes. HSL is stimulated by catecholamine, thyroxine, and glucocorticoid^66^, and in testes, HSL is activated by chorionic gonadotropin. Inactivation of ATGL causes diacylglyceride accumulation in testes^23^, but HSL ablation disables spermatogenesis and causes male infertility^67^. Despite these crucial roles of lipases, the control of lipolysis and even the catalog of lipases (except HSL) are largely unknown in testes. Although lipases can act on broad lipid substrates (e.g., glycerolipids in adipocytes), in Leydig cells, they predominately hydrolyze cholesterol esters to cholesterols for steroidogenesis^68^. Unlike testicular LDs, the LDs in other types of cells including adipocytes were not found to contain so many lipases/esterases and enzymes for glycerolipid and phospholipid metabolism. Likely, testicular LDs need to be accurately modulated by these different enzymes, to facilitate the biosynthesis and hydrolysis of cholesteryl esters and thereby ensure cholesterol supply for steroidogenesis in testes.

The second unique feature is that testicular LDs contained a large number of steroidogenic enzymes such as lanosterol synthase and demethylase, various hydroxysteroid and retinol dehydrogenases, and various glucuronidation enzymes. Currently, steroidogenic enzymes are known to locate in the ER and mitochondrial membranes and in the adjacent cytoplasm, where they catalyze different reactions, their substrates and products being shuttled between these compartments^47^,^69^. The enzymes identified in testicular LDs, such as short-chain dehydrogenase 3^37^,^38^, retinol dehydrogenase 10^36^, 17β-HSD11^41^,^42^, 3β-HSD1^39^,^40^, and NAD(P)H steroid dehydrogenase-like protein^43^,^44^, another 3β-HSD, can translocate from the ER membrane to the LD surface on acyl ester biosynthesis. The substrates, products and metabolites of steroidogenic reactions are mostly insoluble and cannot distribute and move freely in the cytoplasm but instead could be chaperoned and escorted by hydrophobic LDs. Thus, considering that testicular LDs are spatially close to the ER and mitochondria and contain so many steroidogenic enzymes at the oil–water interface, the present data suggests that testicular LDs could be a new compartment for carrying out steroidogenic reactions, more than just a simple pool of cholesterol substrates. At least, testicular LDs could be a chaperone vehicle to facilitate the biosynthesis of hormonal steroids, by transferring insoluble intermediate substrates and products between the mitochondria and the adjacent cytoplasm.

Third, testicular LDs contained large numbers of proteins involved in cellular signaling, chaperon, ubiquination, transport, cytoskeleton and spermatogenesis. Proteins in the GTPase superfamily and Rab GTPase subfamily were particularly abundant. Rab18^53^,^55^ can recruit microtubules and localize between the ER and LDs to facilitate membrane trafficking and lipid exchange^53^,^55^. Ubxd8 and p97/VCP colocalize at the ER-LD interface and promote LD expansion by binding ATGL and inhibiting ATGL-mediated LD lipolysis^49^. Similarly, the vesicle transporters COPI and COPII are membrane bridges between the ER and LDs to deliver and modulate ATGL, Plin2 and Plin3 levels at nascent LDs^60^. Because many of these proteins may exist in large multicomponent complexes, their simultaneous identification from testicular LDs was not surprising. For example, Aup1 localizes to the ER and LDs and contributes to the formation of LDs that may temporarily store misfolded ER proteins under certain conditions^48^,^57^. Actually, Aup1 is a component of the Hrd1–Sel1l ER quality-control complex and physiologically associates with a hundred other proteins^48^. In comparison, testicular LDs contained at least dozens of Aup1-associated proteins^48^, such as Ubxd8, Ubxd2, p97/VCP, Atad3a, Sel1l, Ruvb-like 1, stomatin, ribophorin I, T-complex proteins, epoxide hydrolase, atlastin-3, Acsl3, pyruvate kinase 2/3, and PDI3a. In addition, testicular LDs contained many cytoskeletal proteins, which might not be simply considered contamination. In steroidogenic cells, the LDs and mitochondria are known to tightly attach to the cytoskeleton and intermediate filaments that are thought to mediate transport of cholesterol^70^. An example is vimentin filaments, which bind Plin1 and wrap LDs^12^. Vimentin ablation results in defective steroidogenesis in adrenocortical and granulosa cells^69^. Overall, these findings suggest that testicular LDs could participate initially in cellular signaling, chaperon, ubiquination, transport, cytoskeleton and spermatogenesis.

In summary, testicular LDs could be considered active cellular organelles participating in the regulation of multiple testicular functions. Plins and lipases/esterases/phospholipases could govern accurate control of the biosynthesis and hydrolysis of cholesteryl esters, thus ensuring appropriate cholesteryl ester-triacylglyceride balance and cholesterol supply for steroidogenesis. Notably, the association with various kinds of steroidogenic enzymes suggests that steroidogenic reactions might occur in testicular LDs or the steroidogenic enzymes and products could be transferred through testicular LDs. Because little was known about testicular LD proteins, the investigation of the roles of testicular LDs has been largely restricted to morphological observations. The present finding uncovers the full set of testicular LD proteins, for further examination of the functional roles of testicular LDs and their proteins in steroidogenesis and spermatogenesis in testes.

## Additional Information

**How to cite this article**: Wang, W. *et al*. Proteomic analysis of murine testes lipid droplets. *Sci. Rep*. **5**, 12070; doi: 10.1038/srep12070 (2015).

## Supplementary Material

Supplementary Information

## Figures and Tables

**Table 1:** Proteins associated with testicular lipid droplets (LDs).

Symbol	Protein Name	Remarks^a^	Expectation^b^	GI number
**Group 1: Classic lipid-droplet proteins**				
Plin1	Perilipin 1 (Perilipin)	&^29^; #Adipocyte^29^,^71^. Specific in adipose and steroidogenic cells	3.33E-14	164698413
Plin2	Perilipin-2 (ADRP)	&^29^; #Ubiquitous^11^,^19^,^20^,^28^,^29^,^30^,^40^,^41^,^71^	1.62E-12	116235489
Plin3	Perilipin-3 (TIP47)	&^29^; #Ubiquitous^19^,^29^,^30^,^40^,^71^	1.37E-10	13385312
Plin4	Perilipin-4 (S3-12)	&^29^; #Ubiquitous^28^,^29^,^30^	4.24E-8	157041252
Vim	Vimentin	&^12^; #Ubiquitous^11^,^20^,^28^,^29^,^71^	1.22E-10	31982755
Stom	Stomatin	&^13^; #A431^13^, CHO^28^	2.82E-8	7710018
**Group 2: Lipases**				
HSL	Hormone-sensitive lipase	&^14^; #Adipocyte^29^,^71^, Muscle^30^, Caco-2^40^. Specific in adipose and steroidogenic cells	6.23E-12	87239970
ATGL	Adipose triglyceride lipase	&^23^; #CHO^11^,^28^, Muscle^30^, Coca-2^40^	9.68E-7	254826780
CGI-58	CGI-58 (ATGL coactivator)	&^29^; #Ubiquitous^11^,^28^,^29^,^30^,^40^. α/β-hydrolase	3.29E-13	13385690
Tgh/Ces3	Triacylglycerol hydrolase	&^17^,^18^; #Adipocyte^17^. Testis	1.04E-6	117553604
Mgll	Monoglyceride lipase	#Ovary^20^, Muscle^30^, Liver^19^, Caco-2^39^. Testes	1.72E-9	261878509
Ldah	LD-associated hydrolase (C2orf43)	&^21^,^22^; #Microphage^22^, Caco-2^40^.	2.63E-12	268370116
Lmf2	Lipase maturation factor 2	Testes	7.00E-9	30725786
**Group 3: Glycerolipid metabolism**				
FATP-1	Fatty acid transport protein 1	&^24^; #Ovary^20^. SLC27a1	5.09E-9	6755546
Acsl1	Long-chain acyl-CoA synthetase 1	&^25^; #CHO^11^, Adipocyte^29^, Muscle^30^	9.68E-11	31560705
Acsl3	Long-chain acyl-CoA synthetase 3	&^25^,^26^; #CHO^11^, Adipocyte^29^, Muscle^30^	7.04E-10	209977076
Acsl4	Long-chain acyl-CoA synthetase 4	&^27^; #Ubiquitous^11^,^28^,^29^,^30^,^40^,^71^. Testes	7.69E-13	46518528
Acsl6	Long-chain acyl-CoA synthetase 6		2.00E-10	75992911
Acsbg1	Long-chain acyl-CoA synthetase Acsbg1	Testes	5.03E-13	16716465
Acsvl2	Very long-chain acyl-CoA synthetase 2		1.76E-10	124487285
Acsvl3	Very long-chain acyl-CoA synthetase 3	Testes	4.59E-4	254553374
Acadvl	Very long-chain acyl-CoA dehydrogenase	#Muscle^30^	1.20E-9	23956084
Fasn	Fatty acid synthase	#Ovary^20^	4.20E-8	93102409
Aldh3	Fatty aldehyde dehydrogenase	&^27^,^72^; #Yeast^72^. Microsomal	1.74E-7	75677435
Aldh2	Aldehyde dehydrogenase	#Adipocyte^71^. Mitochondrial	9.73E-9	6753036
Gnpat	Glycerone-phosphate O-acyltransferase	Testes	4.99E-8	160298207
Gpat1	Glycerol-3-phosphate acyltransferase 1	Mitochondrial	1.43E-4	34536827
Gpdh	Glycerol-3-phosphate dehydrogenase	Mitochondrial; sperm capacitation	1.51E-10	224922803
Gk2	Glycerol kinase, testis specific 2	Testis specific	4.74E-10	6754000
Gk5	Glycerol kinase 5		2.39E-8	28893497
Cpt2	Carnitine O-palmitoyltransferase 2	#Muscle^30^	2.81E-10	162138915
Crat	Carnitine O-acetyltransferase		2.18E-6	85662408
Hadha	Trifunctional enzyme subunit α	#Ovary^20^, Muscle^30^	7.94E-10	33859811
Acox3	Acyl-CoA oxidase 3, peroxisomal	Testes	4.17E-5	34328334
Alox12	Arachidonate 12-lipoxygenase		7.88E-10	31542127
**Group 4: Phospholipid metabolism**				
Plb1	Phospholipase B	&^31^; Activated on sperm sterol removal	5.41E-5	194440670
Cpla2	Cytosolic phospholipase A2	&^32^,^33^; #CHO^11^, Muscle^30^. LD formation	2.06E-11	6679369
Pcyt1a	Phosphocholine cytidylyltransferase A	&^34^,^35^; Muscle^30^. LD expansion	1.88E-8	253683458
Pgs1	Phosphatidylglycerophosphate synthase 1	#Muscle^30^. Testes	4.91E-8	110626163
Ddhd1	Phospholipase DDHD1	PA-PLA1	1.20E-5	111955212
Plaa	Phospholipase A2-activating protein		2.27E-5	114431250
Sac1	Phosphatidylinositide phosphatase Sac1		6.66E-9	13507622
Plp	Phosphoinositide lipid phosphatase	Testes	5.48E-8	23956130
Pik3c3	PI3-kinase type 3		4.14E-7	42475974
Pik3r4	PI3-kinase regulatory subunit 4		7.27E-7	124486789
Sphk2	Sphingosine kinase 2		6.26E-8	289191399
**Group 5: Biosynthesis of sterols and hormonal steroids**				
Lss	Lanosterol synthase	&Yeast^27^; #CHO^28^, Adipocyte^29^,^71^, Muscle^30^	3.96E-10	22122469
Cyp51	Lanosterol 14-α demethylase		6.27E-11	71061451
Nsdhl	NAD(P)H steroid dehydrogenase-like	&^43^,^44^; #Ubiquitous^19^,^29^,^30^,^40^.	3.33E-16	31982437
Cyp17a1	17α-hydroxyprogesterone aldolase	Testosterone synthesis	4.74E-9	160948601
Hsd3b1	3β-hydroxysteroid dehydrogenase 1	&^39^,^40^. #Ovary^20^, Caco-2^40^	6.12E-13	6680289
Hsd3b7	3β-hydroxysteroid dehydrogenase 7	#Muscle^30^, Caco-2^39^	1.70E-6	100817048
Hsd17b4	17β-hydroxysteroid dehydrogenase 4	#Muscle^30^	3.66E-9	31982273
Hsd17b7	17β-hydroxysteroid dehydrogenase 7	#CHO^11^,^28^, Adipocyte^29^, Caco-2^40^	1.22E-11	87162470
Hsd17b11	17β-hydroxysteroid dehydrogenase 11	&^41^,^42^; #Muscle^30^, Caco-2^40^	1.08E-11	16716597
Hsdl2	Hydroxysteroid dehydrogenase-like 2		2.93E-5	125656150
Rdh14	Retinol dehydrogenase 14	#Caco-2^40^	3.89E-6	12963791
Rdh10	Retinol dehydrogenase 10	&^36^; #Muscle^30^, Caco-2^40^	4.88E-14	25141231
Aldh1a1	Retinal dehydrogenase 1	Rdh10 counteracted	3.38E-10	85861182
Dhrs3	Short-chain dehydrogenase/reductase 3	&^37^,^38^; #Muscle^30^, Caco-2^40^	1.05E-9	289063391
Dhrs1	Dehydrogenase/reductase SDR member 1	#CHO^11^,^28^, Adipocyte^29^,^71^, Caco-2^40^	4.64E-13	31980844
Dhrsx	Dehydrogenase/reductase X-linked		7.04E-12	124244062
mEH	Epoxide hydrolase 1	#Caco-2^40^. Ephx1	1.60E-10	6753762
Abcd3	ATP-binding cassette transporter D3	Sterol transport in testes	1.54E-8	60218877
Scarb1	Cavenger receptor class B-I	#Ovary^20^. Cholesterol uptake	2.02E-5	14389423
**Group 6: Glucuronidation and glycosylation processes**				
Alg5	DolP-glucosyltransferase	#CHO^11^,^28^	1.71E-8	21728372
Rpn1	Ribophorin I	#CHO^11^, Adipocyte^29^,^71^. OST	8.77E-14	282398108
Stt3a	Oligosaccharyltransferase Stt3a		1.40E-5	148747128
Stt3b	Oligosaccharyltransferase Stt3b		2.71E-9	61651673
Uggt1	UDP–Glc:glycoprotein glucosyltransferase		1.38E-6	236466498
Ugt1a6	UDP-glucuronosyltransferase 1–6	#Caco-2^40^	4.36E-9	33186906
Mettl7a	Methyltransferase-like protein 7A	&^19^,^45^; #CHO^28^, Caco-2^40^. AAM-B	1.24E-10	33563290
CGI-49	CGI-49	#Ubiquitous^11^,^28^,^29^,^30^,^40^	1.20E-11	30520019
Pigt	GPI transamidase component PIG-T	Glycolipid biosynthesis	1.90E-6	120587021
Pigs	GPI transamidase component PIG-S	Complexed with Pigt	2.06E-8	41351529
Dpy19l2	Dpy-19-like protein 2	Spermatogenesis	5.69E-5	261245007
Ganab	α-glucosidase 2	#Ovary^20^, Caco-2^40^	1.39E-7	6679891
Man2a1	α-mannosidase 2		2.52E-7	226246610
Mogs	Mannosyl-oligosaccharide glucosidase		2.53E-8	31981106
Glb1	β-galactosidase		1.82E-5	6753190
Glb1l3	β-galactosidase-1-like protein 3		2.05E-10	164519028
Pcyox**1**	Prenylcysteine oxidase	#Caco-2^40^. Testes	9.00E-9	13385294
**Group 7: Carbohydrate process**				
Slc2a3	Glucose transporter 3		8.33E-12	261862282
Pkm2	Pyruvate kinase 2/3	#CHO^11^, Retina^51^	7.05E-9	31981562
Hk1	Hexokinase-1		1.63E-8	225735584
Hk2	Hexokinase-2		9.10E-7	7305143
Ldha	Lactate dehydrogenase A	#Ovary^20^, Retina^51^. Sperm glycolysis	4.60E-8	6754524
Aldoa	Fructose-bisphosphate aldolase A	#Caco-2^40^. Sperm glycolysis	3.25E-8	293597567
Pfkm	6-phosphofructokinase type A		4.70E-10	254553346
Pfkp	6-phosphofructokinase type C		9.63E-10	9790051
Pygb	Glycogen phosphorylase	Brain form	4.59E-4	24418919
**Group 8: Tricarboxylic acid cycle**				
Cyb5r3	NADH-cytochrome b5 reductase	&^45^; Ubiquitous^11^,^28^,^29^,^40^,^71^. Diaphorase-1	2.33E-14	19745150
Por	NADH P450 oxydoreductase	#Caco-2^40^	5.41E-12	6679421
Ndufs1	Complex I-75kD	#CHO^11^. NADH dehydrogenase	5.21E-10	229892316
Ndufs2	Complex I-49kD		8.05E-9	23346461
Ndufs8	Complex I-23kD		3.20E-9	46195430
Ndufa9	Complex I-39kD		4.67E-9	254692859
Ndufa10	Complex I-42kD		1.26E-9	13195624
Me1	NADP-dependent malic enzyme		1.63E-7	162139827
Uqcrc1	Cytochrome b-c1 complex subunit 1	Complex III	5.08E-12	46593021
COXII	Cytochrome c oxidase subunit II	Complex II	1.05E-13	34538601
Dld	Dihydrolipoamide dehydrogenase	Sperm capacitation	2.07E-6	31982856
Dlst	Dihydrolipoamide S-succinyltransferase	#Ovary^20^	4.69E-9	21313536
Nampt	Nicotinamide phosphoribosyltransferase		1.27E-6	257153454
Sdha	Succinate dehydrogenase subunit A	#Ovary^20^	5.88E-7	54607098
Suclg1	Succinyl-CoA synthase α		8.05E-8	255958286
Glud1	Glutamate dehydrogenase 1	#Retina^51^	4.83E-5	6680027
Aco2	Aconitate hydratase	#Ovary^20^	6.93E-9	18079339
Cs	Citrate synthase	#Ovary^20^	3.15E-6	13385942
Fh1	Fumarate hydratase		2.00E-7	226823367
Mdh2	Malate dehydrogenase	#Ovary^20^, Retina^51^, Caco-2^40^	5.55E-15	31982186
**Group 9: Small GTPases**				
Rab1	Rab1	#CHO^28^, Muscle^30^. Sperm flagella	1.40E-10	6679587
Rab1b	Rab1b	#Muscle^30^. Sperm flagella	1.79E-12	21313162
Rab2a	Rab2a	#CHO^11^,^28^, Muscle^30^	6.09E-9	10946940
Rab2b	Rab2b	#CHO^28^, Muscle^30^	9.39E-9	30525051
Rab4a	Rab4a	#Muscle^30^	6.63E-6	171184402
Rab5a	Rab5a	&^53^; #CHO^11^, Muscle^30^	1.96E-5	13385374
Rab5c	Rab5c	#Ubiquitous^11^,^20^,^28^,^29^,^30^,^71^	9.77E-9	113866024
Rab7	Rab7	#CHO^28^, Adipocyte^29^,^71^, Muscle^30^	9.09E-8	148747526
Rab8a	Rab8a	#Ovary^20^, CHO^11^,^28^, Muscle^30^	6.51E-11	38372905
Rab8b	Rab8b	#Muscle^30^	1.58E-8	27734154
Rab10	Rab10	#CHO^11^,^28^, Muscle^30^	3.33E-6	7710086
Rab11a	Rab11a	&^53^; #CHO^11^,^28^, Adipocyte^29^, Muscle^30^	2.80E-13	31980840
Rab14	Rab14	#CHO^11^,^28^, Adipocyte^29^, Muscle^30^	2.86E-10	18390323
Rab18	Rab18	&^53^,^55^; #CHO^11^,^28^, Adipocyte^29^, Muscle^30^	7.15E-11	30841008
Rab21	Rab21	#Muscle^30^	4.84E-9	33859751
Rab22a	Rab22a	#Muscle^30^	5.22E-9	148747177
Rab31	Rab31	#Muscle^30^	1.10E-7	225579124
Rap1a	Rap1a	#Ovary^20^, Muscle^30^	5.78E-8	21704066
Rap1b	Rap1b	#Muscle^30^, Liver^19^, HuH7^41^	7.85E-9	33859753
Iqgap1	Cdc42-Rac1 effector protein	#Sebocyte^58^	9.31E-8	242332572
Arhgap1	Rho GTPase-activating protein 1	#CHO^11^, Adipocyte^71^. Cdc42 activator	1.93E-7	225543424
Cdc42	Cdc42 GTPase	#Muscle^30^	2.04E-4	6753364
Arl8a	ADP-ribosylation factor-like 8A	#Muscle^30^	6.76E-9	23956194
Arl8b	ADP-ribosylation factor-like 8B	#Muscle^30^. Arf-like GTPase	3.95E-5	13385518
Elmod2	ELMO domain-containing protein 2	&^54^; #Muscle^30^, Caco-2^40^. Arl2 GTPase	7.54E-7	283436077
Ehd1	EH domain-containing protein 1	&^73^; #Ehd2,4 in Muscle^30^. Testilin; Testes .	1.31E-8	7106303
Irgc1	Interferon inducible GTPase 5		5.64E-6	134031980
Atl3	GTPases atlastin-3	#CHO^11^	5.51E-9	254826716
**Group 10: Protein chaperones**				
Hspd1	Heat shock protein 60 kDa	#Ubiquitous^11^,^20^,^30^,^40^	4.06E-11	183396771
Hspa1l	Spermatid-specific HSP70	#Muscle^30^. Spermatogenesis	5.12E-10	124339838
Hspa1b	Heat shock protein 70.1	&Adipocyte^56^; #Ovary^20^, Caco-2^40^	6.59E-5	124339826
Hspa2	Heat shock protein 70.2	#Muscle^30^. Testis specific	7.77E-14	31560686
Hspa4l	Heat shock 70 kDa protein 4 L		2.20E-7	40254361
Hspa8	Heat shock protein cognate 70	#Ubiquitous^20^,^29^,^30^,^40^	2.16E-13	31981690
Hspa5	Glucose-regulated protein 78 kDa	#Ubiquitous^11^,^20^,^28^,^29^,^30^,^40^,^71^. Grp78	1.39E-6	254540166
Hyou1	Hypoxia upregulated protein 1	#Liver^19^	4.61E-12	157951706
Hsp90aa1	Heat shock protein 90-α	#Ovary^20^	7.44E-14	6754254
Hsp90ab1	Heat shock protein 90-β	#CHO^11^, Muscle^30^, Caco-2^40^	1.81E-8	40556608
Hsp90b1	Heat shock protein 90-β member 1	#Muscle^30^, Caco-2^40^	4.73E-8	6755863
Hspa9	Heat shock protein cognate 74	#Muscle^30^, Caco-2^40^	2.39E-9	162461907
Dnajc7	dnaJ (Hsp40) homolog c7	#CHO^11^	1.24E-6	31980994
Dnajc10	dnaJ (Hsp40) homolog c10		5.70E-5	119508443
Dnajc13	dnaJ (Hsp40) homolog c13		8.54E-8	247494234
Pdia1	Protein disulfide-isomerase	#CHO^11^, Caco-2^40^, Liver^19^	3.51E-4	42415475
Pdia3	Protein disulfide-isomerase A3	#Caco-2^40^. Spermatogenesis	1.11E-11	112293264
Pdia4	Protein disulfide-isomerase A4	#Adipocyte^71^, Caco-2^40^	8.73E-8	86198316
Pdilt	Protein disulfide-isomerase Pdilt	Testes specific. fertility	2.23E-10	253735751
Canx	Calnexin	&^19^,^29^. #Ubiquitous^11^,^20^,^29^,^30^,^40^,^71^	6.01E-10	160333216
Calr	Calreticulin	#Liver^19^, Caco-2^40^. Chaperone	4.47E-7	6680836
Tcp1	T-complex protein 1α	Chaperone complex	5.62E-11	110625624
Cct2	T-complex protein 1β (TCP-1β)	#Ovary^20^	6.49E-12	126521835
Cct3	T-complex protein 1γ		1.04E-8	6753320
Cct4	T-complex protein 1 delta		2.23E-8	6753322
Cct5	T-complex protein 1 epsilon		2.39E-8	6671702
Cct6a	T-complex protein 1 zeta		1.22E-6	6753324
Cct7	T-complex protein 1 eta		1.33E-11	238814391
Cct8	T-complex protein 1 theta	Sperm capacitation.	1.32E-7	126723461
Tcp11	T-complex protein 11	Spermatogenesis	1.38E-4	148277067
**Group 11: Ubiquination process**				
Atad3a	AAA ATPase Atad3a	Mitochondrial dynamics	7.30E-5	239985513
Afg3l2	AAA ATPase Afg3l2	AFG3-like protein 2	2.90E-12	110625761
p97/Vcp	AAA ATPase p97 (Vcp)	&^49^,^50^; #Muscle^30^. Binds Ubxd8.	2.22E-15	225543319
Ubxd8	UBX domain-containing protein 8	&^46^,^49^,^50^. #Ubiquitous^11^,^29^,^30^,^40^. Binds Aup1 and Sel1l	1.51E-10	158533976
Ubxd2	UBX domain-containing protein 2	&^50^; #CHO^11^, Caco-2^40^. Ubxn-2, Ubxn-4	9.05E-12	85861252
Aup1	Ancient ubiquitous protein 1	&^48^,^57^; #Ubiquitous^11^,^20^,^28^,^29^,^40^	3.14E-8	90403601
Sel1l	Protein sel-1 homolog 1	Binds Sel1l, Aup1, Ubxd8 and p97	8.53E-12	46309573
Ube1	Ubiquitin-activating enzyme E1		6.61E-9	444189294
Ube3b	Pbiquitin protein ligase E3B		9.08E-10	68533242
Ube4a	Ubiquitination factor E4A		2.33E-8	167736371
Usp7	Ubiquitin specific protease 7		8.83E-6	154146209
Psmd2	26S proteasome regulatory subunit S2	#Ovary^20^	1.71E-8	19882201
Ufl1	E3 UFM1-specific ligase 1	E3 ligase family	6.63E-11	227330590
Fbxl20	F-box/LRR-repeat protein 20	E3 ligase family	2.10E-6	111494221
Bat3	Large proline-rich protein Bat3		2.47E-5	33147082
Cand1	TBP-interacting protein	Cullin-associated	3.11E-14	189409138
Cul3	Cullin-3	E3 ligase family	1.55E-8	7710014
Cul5	Cullin-5	E3 ligase family	6.77E-9	239051067
**Group 12: Transport proteins**				
Sec23a	Protein transport protein Sec23A	&^60^; COPII subunit	1.17E-8	67906177
Sec63	Translocation protein Sec63	Binds Ubxd2	5.26E-6	158937300
Scfd1	Sec1 family domain-containing 1	Vesicle transport	6.41E-7	58037481
Copa	Coatomer (COPI) subunit α	&^59^,^60^; #CHO^11^. COPI-α.	4.77E-8	226823359
Copb	Coatomer subunit β	&^59^,^60^; #CHO^11^.	6.27E-10	15426055
Copg1	Coatomer subunit γ1	&^59^,^60^; #CHO^11^. Testes	6.59E-6	8567338
Copg2	Coatomer subunit γ2	&^59^,^60^; #CHO^11^. Binds CDC42	1.97E-5	8567340
Cog6	COG complex subunit 6	Binds Zw10	3.05E-5	160333744
Zwilch	Zwilch	Zwilch-Zw10 complex	1.89E-6	257153357
Zw10	Zw10	#CHO^11^, Sebocyte^58^	2.42E-8	22165349
Rint1	RAD50-interacting protein 1	Zw10-Sec30-Rint1 complex	1.40E-6	62899067
Trappc8	Trappc8		4.86E-7	291621688
Trappc11	Trappc11	Zw10-Trappc complex	1.61E-6	62241019
Slc18a1	Vesicular amine transporter 1		8.50E-6	33859662
Vps13a	Vacuolar protein sorting 13A	#Muscle^30^	1.78E-12	66392160
Vps13c	Vesicle protein sorting 13C	#Muscle^30^	1.41E-11	122114537
Vps13d	Vesicle protein sorting 13D	#Muscle^30^	5.01E-5	189491889
Vps16	Vesicle protein sorting 16		9.95E-6	254939640
Vps35	Vesicle protein sorting 35	#Ovary^20^	2.79E-8	13928670
Cltc	Clathrin heavy chain 1	#CHO^11^, Muscle^30^	3.56E-9	51491845
Ap1b1	Clathrin adaptor Ap1b1		8.90E-7	88853578
Ap2a1	Clathrin adaptor Ap2a1		5.76E-6	116256510
Ap2b1	Clathrin adaptor Ap2b1		9.22E-7	78711838
Ap2b2	Clathrin adaptor Ap2b2		9.13E-10	163644277
Ncstn	Nicastrin		3.80E-4	224809376
Ncln	Nicastrin-like protein		2.60E-7	33469043
Nomo1	Nicalin-nodal modulator 1		1.74E-8	227908803
Wdr35	WD repeat-containing protein 35		2.23E-8	226958503
Nup93	Nucleoporin 93		1.15E-7	27369533
Nup98	Nucleoporin 98		4.75E-6	39930413
Nup188	Nucleoporin 188		1.84E-4	38678526
Nup210l	Nucleoporin 210 like		6.46E-9	254675162
Kpna3	Importin α4 (karyopherin α3)		2.22E-4	6680596
Kpna6	Importin α7 (karyopherin α6)		2.16E-8	227116300
Kpnb1	Importin β1	#Caco-2^40^	6.26E-8	88014720
Ipo4	Importin-4		2.36E-6	19745156
Ipo5	Importin-5		1.75E-12	29789199
Xpo1	Exportin-1	#Ovary^20^, Sebocyte^58^	1.52E-7	38604071
Xpo2	Exportin-2	#Sebocyte^58^	2.58E-9	12963737
Xpo7	Exportin-7		7.54E-6	12746422
Anxa2	Annexin A2	#Ovary^20^, CHO^11^,^28^, Muscle^30^	4.09E-9	6996913
Anxa6	Annexin A6	#Adipocyte^71^, Muscle^30^, Liver^19^	4.46E-9	158966670
Snx25	Sorting nexin-25	Phospholipid binding	8.97E-9	258613896
**Group 13: Nucleotide-catabolic process**				
Atp5a1	ATP synthase subunit α	#Ovary^20^, CHO^11^. Sperm flagella	2.92E-10	6680748
Atp5b	ATP synthase subunit β	#Ovary^20^, Adipocyte^29^, Caco-2^40^	1.33E-12	31980648
Atp5f1	ATP synthase subunit b		2.21E-8	78214312
Atp1a1	Sodium pump subunit α1	#Caco-2^40^. Spermatogenesis	1.02E-5	21450277
Atp1a4	Sodium pump subunit α4	Spermatogenesis	1.33E-4	226958351
Ctps	CTP synthase		3.24E-11	172072613
Gmps	GMP synthase		4.31E-7	85861218
Umps	UMP synthase		3.43E-8	33859498
Atp6v1a	V-ATPase subunit A	#Ovary^20^	3.49E-7	31560731
Atp6v1h	V-ATPase subunit H		4.70E-6	31981588
Atp13a1	Atp13a1		7.59E-5	283135194
Atp13a2	Atp13a2		6.67E-6	256985106
Atp2a1	SR Ca(2+)-ATPase 1	#Muscle^30^	3.07E-8	36031132
Atp2a2	SR Ca(2+)-ATPase 2	#CHO^11^, Muscle^30^	1.54E-10	6806903
Rent1	ATP-dependent helicase Rent1		4.00E-8	170784813
Eprs	Glutamyl-tRNA synthase		4.54E-7	82617575
Iars2	Isoleucyl-tRNA synthase		6.26E-5	38490690
hnRNPK	hnRNP K		4.75E-6	13384620
Pcbp1	Poly(rC)-binding protein 1		1.95E-8	6754994
Ruvbl1	RuvB-like 1 (AAA ATPase)		5.05E-8	9790083
Eef1a1	Elongation factor 1α1	#CHO^11^, Caco-2^40^	4.29E-10	126032329
Eef2	Elongation factor 2		5.43E-8	33859482
Eif4a2	eIF4A-II		1.57E-9	176865998
Gnb2	G protein β2	#Muscle^30^	1.39E-9	13937391
Map2k2	MAPK/ERK kinase 2	&^33^; #Muscle^30^. Testosterone synthesis	4.88E-8	31560267
Ide	Insulin-degrading enzyme		1.17E-6	121583922
**Group 14: Cytoskeletons**				
Acta1	α-actin		4.11E-13	33563240
Actn1	α-actinin-1	#CHO^11^	3.23E-5	61097906
Myh9	Myosin-9	#Ovary^20^	7.18E-7	114326446
Myh10	Myosin-10	#Ovary^20^	2.03E-7	33598964
Myh11	Myosin-11		2.96E-10	241982716
Myo6	Myosin-6		1.20E-10	261823961
Myo1d	Myosin-1d		3.54E-4	118026911
Myl1	Myosin light chain A1/A2		2.89E-5	29789016
Tuba1a	Tubulin α1A		2.02E-7	6755901
Tuba3a	Tubulin α3A	Testis specific	1.51E-5	6678465
Tubb2a	Tubulin β2A	#Caco-2^40^	9.99E-15	33859488
Tubb4b	Tubulin β4B		6.27E-8	22165384
Tubb3	Tubulin β3		1.85E-8	12963615
Tubb5	Tubulin β5	#Adipocytes^29^	1.40E-9	7106439
Tln1	Talin-1		2.57E-5	227116327
Spna2	Spectrin α2	#Ovary^20^, Liver^19^	9.42E-9	115496850
Cap1	Adenylyl cyclase-associated protein 1	Filament dynamic	7.09E-10	157951604
Ckap4	Cytoskeleton-associated protein 4		3.95E-10	62526118
Armc4	Armadillo repeat-containing protein 4	Outer dynein arms	4.90E-5	124487093
Dnahc8	Dynein heavy chain 8	Testis specific	5.23E-6	153792273
Dnchc1	Dynein heavy chain, cytosolic 1		1.23E-13	134288917
Dnchc2	Dynein heavy chain, cytosolic 2		1.96E-8	72534792
Dnm1l	Dynamin-1-like protein	#Muscle^30^	2.30E-7	71061455
Dnm2	Dynamin-2	LD breakdown	2.19E-6	87299637
**Group 15: Testis specific or spermatogenesis**				
Slc25a5	Adenine nucleotide translocase 2	#Ovary^20^. Spermatogenesis	2.58E-7	22094075
Slc25a31	Adenine nucleotide translocase 4	Testis only, spermatogenesis	3.15E-8	254692892
Acr	Acrosin	Sperm serine proteases	1.54E-6	7304853
Spam1	Sperm-specific Spam1 hyaluronidase	Sperm specific	5.32E-7	120407035
Gapdhs	Spermatogenic cell-specific GAPDH-2	Spermatogenesis	5.05E-7	6679939
Spert	Spermatid-associated protein	Spermatogenesis	5.48E-9	256017220
Spata20	Spermatogenesis-associated protein 20	Spermatogenesis	6.40E-11	46485467
Tcam1	Testicular cell adhesion molecule 1	Testis specific	1.05E-4	145279190
Ift122	Intraflagellar transport protein 122	Flagellar transport	2.24E-5	268370099
Clgn	Calmegin	Spermatogenesis	3.10E-10	86262138
Ace	Angiotensin-converting enzyme	Sperm-zona binding	1.23E-7	33468873
Tfrc	Transferrin receptor	Spermatogenesis	4.35E-4	11596855
Odf2	Outer dense fiber of sperm tails 2	Sperm tails	1.77E-6	295054183
Ddx1	DEAD box protein 1	Germ cell specific	1.32E-9	19527256
Ddx4	DEAD box protein 4	Germ cell specific	9.11E-6	225007636
Bpi	Bactericidal permeability-increasing protein	Testis-specific	2.73E-4	29244434
Piwil1	Piwi-like protein 1	Spermatogenesis	5.07E-9	10946612
Tdrd1	Testis antigen 41.1	Testis-specific	2.13E-8	50355696
Stk31	Serine/threonine-protein kinase 31	Testis-specific	6.56E-5	258613856
Shcbp1	Shc binding protein 1	Testes	8.81E-7	85701672
Dpep3	Dipeptidase 3	Germ cell specific	8.85E-13	21313683
Adam6b	ADAM6b	Testis specific	9.16E-4	57222276
Ppm1j	Protein phosphatase 1J	Germ cell specific.	9.99E-15	114205424
Akap3	A-kinase anchor protein 3	Germ cell specific	9.17E-5	160358791
Akap4	A-kinase anchor protein 4	Spermatid specific	1.52E-5	110347483
Akap12	A-kinase anchor protein 12	Germ cell protein	5.12E-8	13626040
**Group 16: Miscellaneous**				
Alb	Albumin	#Liver^19^	3.67E-8	163310765
Slc3a2	Solute carrier family 3 member 2		8.00E-7	238637277
Pgcp	Plasma glutamate carboxypeptidase		2.83E-8	28570174
Ano10	Anoctamin-10		5.95E-6	30794236
Heatr2	Dynein assembly factor 5		1.65E-9	124486915
Cd109	CD109		9.26E-8	23346525
Aifm2	Apoptosis-inducing factor 2	#Caco-2^40^, HuH7^41^. Testes	2.98E-9	85861162
Api5	Apoptosis inhibitor 5		8.42E-8	94158994
Pdcd6ip	PDCD6-interacting protein	Apoptosis	4.35E-8	258547154
Bbs7	Bardet-Biedl syndrome 7 protein		1.30E-5	170650593
Ttc21b	Tetratricopeptide repeat protein 21B	Ciliary transport	2.04E-8	114158711
Ttc25	Tetratricopeptide repeat protein 25		1.15E-4	124358957
Ttc39b	Tetratricopeptide repeat protein 39B		2.05E-5	58037187
Tom70	Mitochondrial import receptor Tom70	Ttc domain	9.95E-4	27552760
Mtch2	Mitochondrial carrier homolog 2		5.64E-7	9790055
Lamp2	Lysosome membrane protein 2		6.09E-9	6680878
Ermp1	ER metallopeptidase 1		2.27E-6	124487057
Fam79a	Fam79a		7.75E-14	21312776
Fam91a1	Fam91a1		7.00E-7	112817622
Fam129a	Fam129a	Niban	3.82E-7	241982745
Mic60	Mic60		1.09E-9	70608131
Stim1	Stromal interaction molecule 1		3.04E-4	31981983
Nbas	Neuroblastoma-amplified protein	#CHO^11^,^28^	2.92E-10	255003837
Lrrc40	Leucine rich repeat containing 40		4.43E-6	31541911
Pdxdc1	Pdxdc1		4.66E-8	88758582
Gcn1l1	Gcn1l1		1.28E-7	112807186
Ilvbl	ilvB-like protein	Acetolactate synthase	4.24E-9	30424591
Trim27	Zinc finger protein RFP		2.19E-6	125347389
Srp68	Signal recognition particle 68		2.64E-4	47271535
Tm9sf2	Transmembrane 9 superfamily member 2		2.70E-8	188528894
unknown	RIKEN cDNA 4732456N10 gene		1.91E-7	269914154

A total of 337 proteins were identified from murine testicular LDs by mass spectrometry; 144 identified proteins had been previously detected in LD proteomic studies and are labeled with “#” and citations to annotate the tissue or cell source of the LDs. A total of 44 proteins had been previously confirmed in LDs by microscopy and are labeled with “&”.

^a^Comparison with the reference data involved manual inspection of the GI number and then the standard names of proteins identified in the present and previous proteomic studies.

^b^The expectation value is a statistical term that allows for comparison of the reliability of results. Protein identifications were based on both the expectation value (<10^−4^) and the quality of MS/MS spectra of peptide fragments (>3) identified. Low expectation values correspond to confident identifications.

## References

[b1] Neaves W. B. Changes in testicular leydig cells and in plasma testosterone levels among seasonally breeding rock hyrax. Biol Reprod 8, 451–466 (1973). 4710785 10.1093/biolreprod/8.4.451

[b2] Mori H. & Christensen A. K. Morphometric analysis of Leydig cells in the normal rat testis. J Cell Biol 84, 340–354 (1980). 6991510 10.1083/jcb.84.2.340 PMC2110560

[b3] Mori H., Hiromoto N., Nakahara M. & Shiraishi T. Stereological analysis of Leydig cell ultrastructure in aged humans. The Journal of clinical endocrinology and metabolism 55, 634–641 (1982). 7107810 10.1210/jcem-55-4-634

[b4] Morris M. D. & Chaikoff I. L. The origin of cholesterol in liver, small intestine, adrenal gland, and testis of the rat: dietary versus endogenous contributions. J Biol Chem 234, 1095–1097 (1959). 13654326

[b5] Freeman D. A. & Ascoli M. Studies on the source of cholesterol used for steroid biosynthesis in cultured Leydig tumor cells. J Biol Chem 257, 14231–14238 (1982). 7142204

[b6] Ariyaratne H. B. & Chamindrani Mendis-Handagama S. Changes in the testis interstitium of Sprague Dawley rats from birth to sexual maturity. Biol Reprod 62, 680–690 (2000). 10684810 10.1095/biolreprod62.3.680

[b7] Paniagua R., Rodriguez M. C., Nistal M., Fraile B. & Amat P. Changes in the lipid inclusion/Sertoli cell cytoplasm area ratio during the cycle of the human seminiferous epithelium. J Reprod Fertil 80, 335–341 (1987). 3037076 10.1530/jrf.0.0800335

[b8] Kerr J. B. & De Kretser D. M. Cyclic variations in Sertoli cell lipid content throughout the spermatogenic cycle in the rat. J Reprod Fertil 43, 1–8 (1975). 1127625 10.1530/jrf.0.0430001

[b9] Brasaemle D. L. The perilipin family of structural lipid droplet proteins: stabilization of lipid droplets and control of lipolysis. J Lipid Res 48, 2547–2559 (2007). 17878492 10.1194/jlr.R700014-JLR200

[b10] Ding Y. . Isolating lipid droplets from multiple species. Nat Protoc 8, 43–51 (2013). 23222457 10.1038/nprot.2012.142

[b11] Bartz R. . Dynamic activity of lipid droplets: protein phosphorylation and GTP-mediated protein translocation. J Proteome Res 6, 3256–3265 (2007). 17608402 10.1021/pr070158j

[b12] Heid H. . On the formation of lipid droplets in human adipocytes: the organization of the perilipin-vimentin cortex. PLoS One 9, e90386 (2013). 24587346 10.1371/journal.pone.0090386 PMC3938729

[b13] Umlauf E. . Association of stomatin with lipid bodies. J Biol Chem 279, 23699–23709 (2004). 15024010 10.1074/jbc.M310546200

[b14] Sztalryd C. . Perilipin A is essential for the translocation of hormone-sensitive lipase during lipolytic activation. J Cell Biol 161, 1093–1103 (2003). 12810697 10.1083/jcb.200210169 PMC2172984

[b15] Hsieh K. . Perilipin family members preferentially sequester to either triacylglycerol-specific or cholesteryl-ester-specific intracellular lipid storage droplets. J Cell Sci 125, 4067–4076 (2012). 22685330 10.1242/jcs.104943 PMC3482316

[b16] Schweiger M. . Adipose triglyceride lipase and hormone-sensitive lipase are the major enzymes in adipose tissue triacylglycerol catabolism. J Biol Chem 281, 40236–40241 (2006). 17074755 10.1074/jbc.M608048200

[b17] Soni K. G. . Carboxylesterase 3 (EC 3.1.1.1) is a major adipocyte lipase. J Biol Chem 279, 40683–40689 (2004). 15220344 10.1074/jbc.M400541200

[b18] Wang H. . Altered lipid droplet dynamics in hepatocytes lacking triacylglycerol hydrolase expression. Mol Biol Cell 21, 1991–2000 (2010). 20410140 10.1091/mbc.E09-05-0364 PMC2883943

[b19] Turro S. . Identification and characterization of associated with lipid droplet protein 1: A novel membrane-associated protein that resides on hepatic lipid droplets. Traffic 7, 1254–1269 (2006). 17004324 10.1111/j.1600-0854.2006.00465.x

[b20] Khor V. K. . The proteome of cholesteryl-ester-enriched versus triacylglycerol-enriched lipid droplets. PLoS One 9, e105047 (2014). 25111084 10.1371/journal.pone.0105047 PMC4128735

[b21] Thiel K. . The evolutionarily conserved protein CG9186 is associated with lipid droplets, required for their positioning and for fat storage. J Cell Sci 126, 2198–2212 (2013). 23525007 10.1242/jcs.120493 PMC3880856

[b22] Goo Y. H., Son S. H., Kreienberg P. B. & Paul A. Novel lipid droplet-associated serine hydrolase regulates macrophage cholesterol mobilization. Arterioscler Thromb Vasc Biol 34, 386–396 (2014). 24357060 10.1161/ATVBAHA.113.302448 PMC3952429

[b23] Zimmermann R. . Fat mobilization in adipose tissue is promoted by adipose triglyceride lipase. Science 306, 1383–1386 (2004). 15550674 10.1126/science.1100747

[b24] Xu N. . The FATP1-DGAT2 complex facilitates lipid droplet expansion at the ER-lipid droplet interface. J Cell Biol 198, 895–911 (2012). 22927462 10.1083/jcb.201201139 PMC3432760

[b25] Wilfling F. . Triacylglycerol synthesis enzymes mediate lipid droplet growth by relocalizing from the ER to lipid droplets. Dev Cell 24, 384–399 (2013). 23415954 10.1016/j.devcel.2013.01.013 PMC3727400

[b26] Poppelreuther M. . The N-terminal region of acyl-CoA synthetase 3 is essential for both the localization on lipid droplets and the function in fatty acid uptake. J Lipid Res 53, 888–900 (2012). 22357706 10.1194/jlr.M024562 PMC3329388

[b27] Natter K. . The spatial organization of lipid synthesis in the yeast Saccharomyces cerevisiae derived from large scale green fluorescent protein tagging and high resolution microscopy. Mol Cell Proteomics 4, 662–672 (2005). 15716577 10.1074/mcp.M400123-MCP200

[b28] Liu P. . Chinese hamster ovary K2 cell lipid droplets appear to be metabolic organelles involved in membrane traffic. J Biol Chem 279, 3787–3792 (2004). 14597625 10.1074/jbc.M311945200

[b29] Brasaemle D. L., Dolios G., Shapiro L. & Wang R. Proteomic analysis of proteins associated with lipid droplets of basal and lipolytically stimulated 3T3-L1 adipocytes. J Biol Chem 279, 46835–46842 (2004). 15337753 10.1074/jbc.M409340200

[b30] Zhang H. . Proteome of skeletal muscle lipid droplet reveals association with mitochondria and apolipoprotein a-I. J Proteome Res 10, 4757–4768 (2011). 21870882 10.1021/pr200553c

[b31] Selvaraju K., Rajakumar S. & Nachiappan V. Identification of a phospholipase B encoded by the LPL1 gene in Saccharomyces cerevisiae. Biochim Biophys Acta 1842, 1383–1392 (2014). 25014274 10.1016/j.bbalip.2014.06.013

[b32] Wooten R. E. . Novel translocation responses of cytosolic phospholipase A2alpha fluorescent proteins. Biochim Biophys Acta 1783, 1544–1550 (2008). 18406359 10.1016/j.bbamcr.2008.03.008 PMC2474710

[b33] Yu W. . Co-compartmentalization of MAP kinases and cytosolic phospholipase A2 at cytoplasmic arachidonate-rich lipid bodies. Am J Pathol 152, 759–769 (1998). 9502418 PMC1858398

[b34] Krahmer N. . Phosphatidylcholine synthesis for lipid droplet expansion is mediated by localized activation of CTP:phosphocholine cytidylyltransferase. Cell Metab 14, 504–515 (2011). 21982710 10.1016/j.cmet.2011.07.013 PMC3735358

[b35] Guo Y. . Functional genomic screen reveals genes involved in lipid-droplet formation and utilization. Nature 453, 657–661 (2008). 18408709 10.1038/nature06928 PMC2734507

[b36] Jiang W. & Napoli J. L. The retinol dehydrogenase Rdh10 localizes to lipid droplets during acyl ester biosynthesis. J Biol Chem 288, 589–597 (2013). 23155051 10.1074/jbc.M112.402883 PMC3537056

[b37] Deisenroth C., Itahana Y., Tollini L., Jin A. & Zhang Y. p53-Inducible DHRS3 is an endoplasmic reticulum protein associated with lipid droplet accumulation. J Biol Chem 286, 28343–28356 (2011). 21659514 10.1074/jbc.M111.254227 PMC3151078

[b38] Adams M. K., Belyaeva O. V., Wu L. & Kedishvili N. Y. The retinaldehyde reductase activity of DHRS3 is reciprocally activated by retinol dehydrogenase 10 to control retinoid homeostasis. J Biol Chem 289, 14868–14880 (2014). 24733397 10.1074/jbc.M114.552257 PMC4031538

[b39] Beilstein F., Bouchoux J., Rousset M. & Demignot S. Proteomic analysis of lipid droplets from Caco-2/TC7 enterocytes identifies novel modulators of lipid secretion. PLoS One 8, e53017 (2013). 23301014 10.1371/journal.pone.0053017 PMC3534623

[b40] Bouchoux J. . The proteome of cytosolic lipid droplets isolated from differentiated Caco-2/TC7 enterocytes reveals cell-specific characteristics. Biol Cell 103, 499–517 (2011). 21787361 10.1042/BC20110024 PMC3181828

[b41] Fujimoto Y. . Identification of major proteins in the lipid droplet-enriched fraction isolated from the human hepatocyte cell line HuH7. Biochim Biophys Acta 1644, 47–59 (2004). 14741744 10.1016/j.bbamcr.2003.10.018

[b42] Horiguchi Y., Araki M. & Motojima K. Identification and characterization of the ER/lipid droplet-targeting sequence in 17beta-hydroxysteroid dehydrogenase type 11. Arch Biochem Biophys 479, 121–130 (2008). 18804447 10.1016/j.abb.2008.08.020

[b43] Ohashi M., Mizushima N., Kabeya Y. & Yoshimori T. Localization of mammalian NAD(P)H steroid dehydrogenase-like protein on lipid droplets. J Biol Chem 278, 36819–36829 (2003). 12837764 10.1074/jbc.M301408200

[b44] Caldas H. & Herman G. E. NSDHL, an enzyme involved in cholesterol biosynthesis, traffics through the Golgi and accumulates on ER membranes and on the surface of lipid droplets. Hum Mol Genet 12, 2981–2991 (2003). 14506130 10.1093/hmg/ddg321

[b45] Zehmer J. K., Bartz R., Liu P. & Anderson R. G. Identification of a novel N-terminal hydrophobic sequence that targets proteins to lipid droplets. J Cell Sci 121, 1852–1860 (2008). 18477614 10.1242/jcs.012013 PMC2849272

[b46] Zehmer J. K. . Targeting sequences of UBXD8 and AAM-B reveal that the ER has a direct role in the emergence and regression of lipid droplets. J Cell Sci 122, 3694–3702 (2009). 19773358 10.1242/jcs.054700 PMC2758803

[b47] Ritter J. K. Roles of glucuronidation and UDP-glucuronosyltransferases in xenobiotic bioactivation reactions. Chem Biol Interact 129, 171–193 (2000). 11154740 10.1016/s0009-2797(00)00198-8

[b48] Klemm E. J., Spooner E. & Ploegh H. L. Dual role of ancient ubiquitous protein 1 (AUP1) in lipid droplet accumulation and endoplasmic reticulum (ER) protein quality control. J Biol Chem 286, 37602–37614 (2011). 21857022 10.1074/jbc.M111.284794 PMC3199505

[b49] Olzmann J. A., Richter C. M. & Kopito R. R. Spatial regulation of UBXD8 and p97/VCP controls ATGL-mediated lipid droplet turnover. Proc Natl Acad Sci U.S.A. 110, 1345–1350 (2013). 23297223 10.1073/pnas.1213738110 PMC3557085

[b50] Suzuki M. . Derlin-1 and UBXD8 are engaged in dislocation and degradation of lipidated ApoB-100 at lipid droplets. Mol Biol Cell 23, 800–810 (2012). 22238364 10.1091/mbc.E11-11-0950 PMC3290640

[b51] Orban T., Palczewska G. & Palczewski K. Retinyl ester storage particles (retinosomes) from the retinal pigmented epithelium resemble lipid droplets in other tissues. J Biol Chem 286, 17248–17258 (2011). 21454509 10.1074/jbc.M110.195198 PMC3089567

[b52] Almahbobi G., Williams L. J., Han X. G. & Hall P. F. Binding of lipid droplets and mitochondria to intermediate filaments in rat Leydig cells. J Reprod Fertil 98, 209–217 (1993). 8345465 10.1530/jrf.0.0980209

[b53] Liu P. . Rab-regulated interaction of early endosomes with lipid droplets. Biochim Biophys Acta 1773, 784–793 (2007). 17395284 10.1016/j.bbamcr.2007.02.004 PMC2676670

[b54] East M. P., Bowzard J. B., Dacks J. B. & Kahn R. A. ELMO domains, evolutionary and functional characterization of a novel GTPase-activating protein (GAP) domain for Arf protein family GTPases. J Biol Chem 287, 39538–39553 (2012). 23014990 10.1074/jbc.M112.417477 PMC3501052

[b55] Martin S., Driessen K., Nixon S. J., Zerial M. & Parton R. G. Regulated localization of Rab18 to lipid droplets: effects of lipolytic stimulation and inhibition of lipid droplet catabolism. J Biol Chem 280, 42325–42335 (2005). 16207721 10.1074/jbc.M506651200

[b56] Jiang H., He J., Pu S., Tang C. & Xu G. Heat shock protein 70 is translocated to lipid droplets in rat adipocytes upon heat stimulation. Biochim Biophys Acta 1771, 66–74 (2007). 17175194 10.1016/j.bbalip.2006.10.004

[b57] Jo Y., Hartman I. Z. & DeBose-Boyd R. A. Ancient ubiquitous protein-1 mediates sterol-induced ubiquitination of 3-hydroxy-3-methylglutaryl CoA reductase in lipid droplet-associated endoplasmic reticulum membranes. Mol Biol Cell 24, 169–183 (2013). 23223569 10.1091/mbc.E12-07-0564 PMC3564538

[b58] Dahlhoff M. . Characterization of the sebocyte lipid droplet proteome reveals novel potential regulators of sebaceous lipogenesis. Exp Cell Res 332, 146–155 (2015). 25523620 10.1016/j.yexcr.2014.12.004

[b59] Wilfling F. . Arf1/COPI machinery acts directly on lipid droplets and enables their connection to the ER for protein targeting. Elife 3, e01607 (2014). 24497546 10.7554/eLife.01607 PMC3913038

[b60] Soni K. G. . Coatomer-dependent protein delivery to lipid droplets. J Cell Sci 122, 1834–1841 (2009). 19461073 10.1242/jcs.045849 PMC2684835

[b61] Zehmer J. K. . A role for lipid droplets in inter-membrane lipid traffic. Proteomics 9, 914–921 (2009). 19160396 10.1002/pmic.200800584 PMC2676673

[b62] Nagayama M., Uchida T. & Gohara K. Temporal and spatial variations of lipid droplets during adipocyte division and differentiation. J Lipid Res 48, 9–18 (2007). 17057226 10.1194/jlr.M600155-JLR200

[b63] Xu G. . Post-translational regulation of adipose differentiation-related protein by the ubiquitin/proteasome pathway. J Biol Chem 280, 42841–42847 (2005). 16115879 10.1074/jbc.M506569200

[b64] Lyu Y. . Defective differentiation of adipose precursor cells from lipodystrophic mice lacking perilipin 1. PLoS One 10, e0117536 (2015). 25695774 10.1371/journal.pone.0117536 PMC4335001

[b65] He J. . Calyculin and okadaic acid promote perilipin phosphorylation and increase lipolysis in primary rat adipocytes. Biochim Biophys Acta 1761, 247–255 (2006). 16545598 10.1016/j.bbalip.2006.02.001

[b66] Xu C. . Direct effect of glucocorticoids on lipolysis in adipocytes. Mol Endocrinol 23, 1161–1170 (2009). 19443609 10.1210/me.2008-0464 PMC5419195

[b67] Osuga J. . Targeted disruption of hormone-sensitive lipase results in male sterility and adipocyte hypertrophy, but not in obesity. Proc. Natl. Acad. Sci. U.S.A 97, 787–792 (2000). 10639158 10.1073/pnas.97.2.787 PMC15409

[b68] Freeman D. A. Regulation of the cholesterol ester cycle of cultured Leydig tumor cells. Eur J Biochem 164, 351–356 (1987). 3032615 10.1111/j.1432-1033.1987.tb11065.x

[b69] Kraemer F. B., Khor V. K., Shen W. J. & Azhar S. Cholesterol ester droplets and steroidogenesis. Mol Cell Endocrinol 371, 15–19 (2012). 23089211 10.1016/j.mce.2012.10.012 PMC3584206

[b70] Hall P. F. & Almahbobi G. Roles of microfilaments and intermediate filaments in adrenal steroidogenesis. Microsc Res Tech 36, 463–479 (1997). 9142693 10.1002/(SICI)1097-0029(19970315)36:6<463::AID-JEMT4>3.0.CO;2-J

[b71] Cho S. Y. . Identification of mouse Prp19p as a lipid droplet-associated protein and its possible involvement in the biogenesis of lipid droplets. J Biol Chem 282, 2456–2465 (2007). 17118936 10.1074/jbc.M608042200

[b72] Currie E. . High confidence proteomic analysis of yeast LDs identifies additional droplet proteins and reveals connections to dolichol synthesis and sterol acetylation. J Lipid Res 55, 1465–1477 (2014). 24868093 10.1194/jlr.M050229 PMC4076087

[b73] Naslavsky N., Rahajeng J., Rapaport D., Horowitz M. & Caplan S. EHD1 regulates cholesterol homeostasis and lipid droplet storage. Biochem Biophys Res Commun 357, 792–799 (2007). 17451652 10.1016/j.bbrc.2007.04.022 PMC1978283

