# Supplementary material for: Proteomic analysis of murine testes lipid droplets
Source: Sci Rep. 2015 Jul 10;5:12070. doi: 10.1038/srep12070 (PMC4498221; doi:10.1038/srep12070)
Supplement: Supplementary Information [file srep12070-s1.pdf]

Proteomic analysis of murine testes lipid droplets

Weiyi Wang [1], Suning Wei [1], Linghai Li [2], Xueying Su [1], Congkuo Du [1], Fengjuan Li [1], Bin Geng [1], Pingsheng Liu [2§], and Guoheng Xu [1§]

[1] Department of Physiology and Pathophysiology, School of Basic Medical Sciences, Peking University, Beijing 100191, China.
[2] National Laboratory of Biomacromolecules, Institute of Biophysics, Chinese Academy of Sciences, Beijing, 100101, China.

Running title: Proteome of testicular lipid droplets

[§]**Address correspondence to:** Guoheng Xu, Email: xug@bjmu.edu.cn
Or to: Pingsheng Liu, Email: pliu@ibp.ac.cn

Supplementary Figure S1. Full-length blots of proteins and silver stained gel shown in Figure 4

The fractions of lipid droplet (LD), total membrane (TM), cytosol (Cyto), and post-nuclear supernatant (PNS) were prepared from mice testes as described in Methods. An equal amount proteins extracted from different fractions were separated by SDS-PAGE and underwent immunoblotting with primary antibodies indicated. The full-length blots of proteins were derived from the sample or different samples that were processed in parallel. A representative silver-stained gel showed the equivalent protein loads.

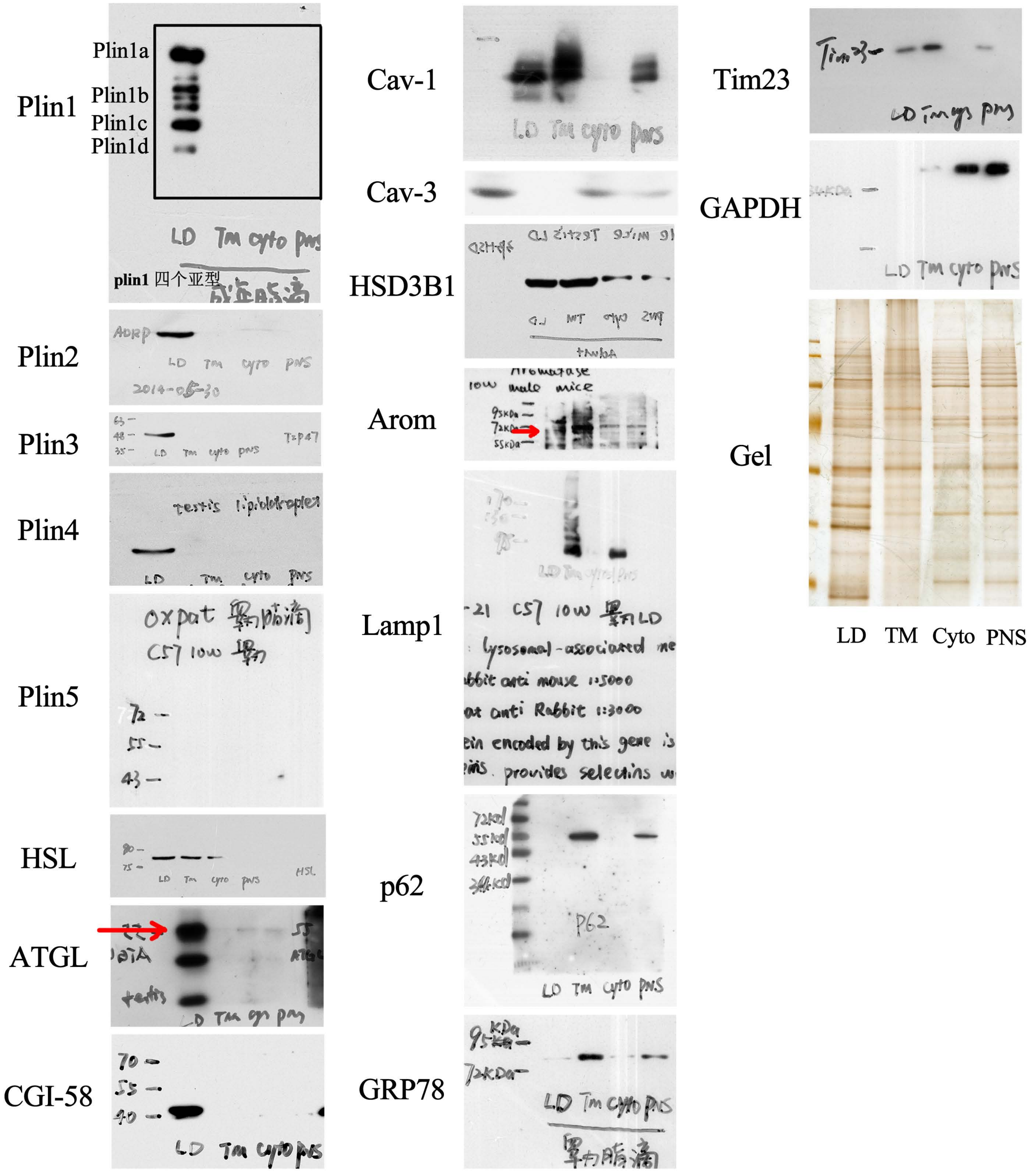
Plin1
Plin1a
Plin1b
Plin1c
Plin1d
plin1 四个亚型
Plin2
Plin3
Plin4
Plin5
HSL
ATGL
CGI-58
Cav-1
Cav-3
HSD3B1
Arom
Lamp1
p62
GRP78
Tim23
GAPDH
Gel
LD
TM
Cyto
PNS
